# Exploring hemoglobin dynamics and scavenging mechanisms in preterm infants and preterm rabbits with cerebral intraventricular hemorrhage

**DOI:** 10.1038/s41390-025-04556-2

**Published:** 2025-11-14

**Authors:** Amanda Kristiansson, Helena Karlsson, Suvi Vallius, Niklas Ortenlöf, Claes Ekström, Katarzyna Wiatrowska, Valérie Verdon, Sandra Mena Perez, Kirsten Guse, Thomas Gentinetta, Markus Brechmann, David Ley, Magnus Gram

**Affiliations:** 1 Department of Clinical Sciences Lund, Pediatrics, Lund University, Lund, Sweden; 2 Department of Neonatology, Skåne University Hospital, Lund, Sweden; 3 The Sahlgrenska Centre for Pediatric Ophthalmology Research, Department of Clinical Neuroscience, Institute of Neuroscience and Physiology, Sahlgrenska Academy, University of Gothenburg, Gothenburg, Sweden; 4 Department of Pathology, Boston Children’s Hospital and Harvard Medical School, Boston, MA USA; 5 CSL Behring, CSL Biologics Research Center, Bern, Switzerland; 6 Swiss Institute for Translational and Entrepreneurial Medicine, Sitem-insel, Bern, Switzerland; 7 CSL Innovation GmbH, Marburg, Germany; 8 Department of Biomedical Science, Faculty of Health and Society, Biofilms-Research Center for Biointerfaces, Malmö University, Malmö, Sweden

## Abstract

**Background:**

Intraventricular hemorrhage (IVH) is a leading cause of neurodevelopmental impairment in preterm infants, driven by vascular rupture, intraventricular blood accumulation, and secondary injury. Hemolysis releases extracellular hemoglobin (Hb) into the cerebrospinal fluid (CSF), where it promotes oxidative stress, inflammation, and cytotoxicity. The endogenous Hb-scavenging protein haptoglobin (Hp) may mitigate Hb-mediated injury, but its capacity in the immature brain remains unclear. This study aimed to characterize Hb and Hp dynamics in the CSF of preterm infants with severe IVH and to evaluate the translational utility of a preterm rabbit glycerol-induced IVH model.

**Methods:**

Fourteen infants with grade III IVH or periventricular hemorrhagic infarction were enrolled in a prospective study. CSF was collected via ventricular access devices and analyzed alongside samples from rabbit pups with induced IVH.

**Results:**

Infants showed markedly elevated CSF Hb (mean ± SEM: 133.3 ± 50.1 µmol/L, heme equivalents) and low Hp (0.87 ± 0.15 µg/mL). Similar patterns were observed in IVH-exposed rabbit pups, with elevated Hb and significantly reduced CSF Hp compared to non-IVH controls.

**Conclusion:**

These findings indicate a persistent deficit in endogenous Hb clearance following IVH and demonstrate that the rabbit pup model closely reflects the human condition, making it well-suited for preclinical evaluation of Hb-scavenging therapies.

**Impact:**

- The aim of this study was to characterize the dynamics of hemoglobin and haptoglobin in the cerebrospinal fluid of preterm infants with severe IVH and to evaluate the translational validity of a preterm rabbit pup IVH model.
- By directly comparing hemoglobin and haptoglobin concentrations across species, this study revealed a profound imbalance between hemoglobin burden and scavenging capacity, highlighting a persistent deficit in endogenous clearance.
- These findings provide a strong rationale for therapeutic strategies targeting hemoglobin clearance in preterm IVH and support the use of the rabbit pup IVH model for translational studies.

## Introduction

Globally, prematurity is the primary cause of childhood morbidity and mortality.^1^ A key contributor to long-term neurodevelopmental impairment in preterm infants is cerebral intraventricular hemorrhage (IVH).^2^ In extremely preterm (EPT, born before gestational week 28) infants, 30–35% develop IVH whereof 16% of these lesions are severe (grade III or IV).^3^ Although survival of EPT infants is increasing, the incidence of neurodevelopmental impairment in survivors remains high,^4,5^ including conditions such as cerebral palsy and mental retardation.^6,7^ There is currently no approved pharmaceutical therapy to prevent or treat IVH in preterm infants; however, clinical trials are evaluating pharmacologic interventions such as erythropoietin (EPO; e.g., EpoRepair, NCT02076373) alone or combined with melatonin (NCT05617833).^8,9^ Until effective treatments are established, current clinical practice focuses on cerebrospinal fluid (CSF) drainage to limit the degree of post-hemorrhagic ventricular dilatation (PHVD).^10,11^

The progression of IVH in the preterm infants is initiated by the rupture of immature vasculature in the germinal matrix (GM),^12^ leading to a buildup of blood within the ventricles, which disrupts normal anatomy and increases the intraventricular pressure. In the subsequent pathophysiological cascade, cell-free hemoglobin (Hb) is released into the CSF following hemolysis.^13^ Cell-free Hb, where each Hb tetramer contains four heme groups, is highly susceptible to oxidative modification and oxidation of Hb to metHb destabilizes the globin structure, promoting heme liberation. Free heme is a potent pro-oxidant, it catalyzes the release of redox-active iron, drives the formation of reactive oxygen species (ROS), and can intercalate into cell membranes, thereby compromising their integrity.^14,15^ As suggested by previous studies, these metabolites act as mediators to severe cerebral damage, hypomyelination and gliosis by initiating inflammation, oxidative stress, and apoptosis.^13,16–20^ In the immature brain, injury following IVH predominantly affects periventricular regions, particularly the GM, ventricular walls, and subventricular zone, which are highly susceptible to oxidative damage due to their rapid growth and relatively limited antioxidant defenses.^21–23^

Further showing the importance of Hb toxicity in IVH, glial cells exposed to hemorrhagic CSF or oxidized Hb in vitro displayed an increase in ROS production, pro-inflammatory response and cytoskeletal disintegration. In addition, co-administration of human haptoglobin (Hp), an endogenous Hb-scavenger, was found to partially block the damaging effects of hemorrhagic CSF and oxidized Hb.^24^ Moreover, evaluation of intraventricularly administered Hp in a preterm rabbit pup model of IVH showed a co-localization with widespread cell-free Hb resulting from the bleeding.^19^ Based on this, it has been proposed that the administration of human Hp, aiming to minimize the spontaneous autoxidation of cell-free Hb and release of heme, may provide therapeutic benefit in preterm infants with IVH.

In this study, we aimed to further explore the scientific rationale of using Hp as a treatment for preterm IVH by characterizing the Hb levels and Hb-scavenging capacity in preterm infants suffering from IVH, and in the preterm rabbit pup model of glycerol-induced IVH. The preterm rabbit pup model of IVH has proved a valuable model for the study of preterm IVH/GM hemorrhage (GMH) of the preterm infant since developed in the 1980s.^25–29^ Preterm rabbit pups respond to the glycerol-induced hyperosmolarity with perturbations of cerebral blood flow resulting in mechanical stress leading to vessel rupture and bleeding into the ventricles,^25,28^ an etiology that resembles the pathophysiology in human preterm infants developing IVH/GMH.^26^ Although the model is well-established, characterization of endogenous Hb-scavenging has not yet been described. Based on this, the main objective of this study was to investigate the clinical translatability of Hb-scavenging in the preterm rabbit pup model of glycerol-induced IVH.

## Methods

### Patient sampling

**Fig. 1:**
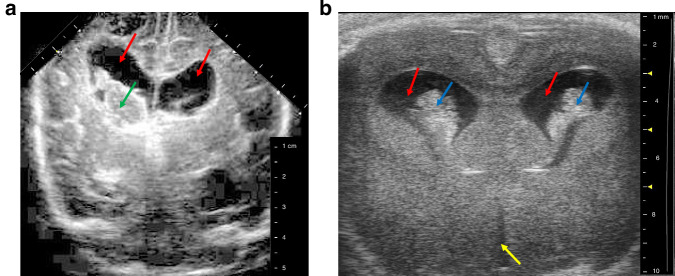
High-frequency ultrasound of intraventricular hemorrhage (IVH) in an infant and a preterm rabbit pup. Coronal images of an infant suffering from IVH (**a**) and preterm rabbit pup with IVH (**b**). Echogenic material within the lateral ventricles was used to determine presence of IVH and assign group classification. Images are provided for illustrative purposes only. Scale bars indicate cm in (**a**) and mm in (**b**). Red arrow = lateral ventricle, green arrow = intraventricular blood clot, blue arrow = choroid plexus with intraventricular blood clot and yellow arrow = third ventricle.

The study of preterm infants with IVH was approved by the Ethical Committee on Human Subjects in Malmö/Lund (Dnr 2009/402). The study was performed as a prospective observational study at a level III NICU, Skåne University Hospital, Lund, Sweden. Fourteen preterm infants with a mean (standard deviation, SD) gestational age at birth of 26.6 (2.4) weeks and a birthweight of 1046 (384) g were recruited for the study. All infants developed either IVH grade III (*N* = 8) or periventricular hemorrhagic infarction (*N* = 6), as diagnosed with bedside ultrasound (Fig. 1a). All infants received a neurosurgically inserted intraventricular reservoir (ventricular access device, VAD) enabling CSF withdrawal. At withdrawal, CSF was collected in a centrifugation tube without any additive, centrifuged 500–1000 x *g* for 10 min, and afterwards the supernatant was transferred to a separate tube and frozen and stored in −80 °C until further analysis.

### Hb analysis of patient CSF

Quantification of Hb in CSF samples was performed by deconvolution of absorption spectra (350-650 nm) measured in a Lunatic spectrophotometer (Unchained Labs, Pleasanton, CA). For this, extinction curves for the individual substances with known concentrations were fitted to the extinction curve of CSF using a nonnegative least squares algorithm as described previously.^30^

### Hp analysis of patient CSF

Absolute protein quantitation of Hp, was achieved by liquid chromatography coupled with a mass spectrometry (LC-MS/MS) approach, using a multiple reaction monitoring (MRM) method following an enzymatic digestion of CSF samples. Prior to sample analysis the method was qualified, fulfilling all acceptance criteria for a bioanalytical method and in accordance with regulatory guidelines.^31,32^

Artificial, synthetic CSF (product: OH1030-S, BioIVT, Westbury, New York) was used as surrogate matrix for calibration curve samples preparation, whereas pooled human CSF (Innovative Research, Novi, Michigan) was used for the preparation of quality control (QC) samples. Calibration curve samples and QC samples were prepared as follows: A serial dilution of spiked plasma-derived proteins, human Hp 1–1 (Behring R&D, Kankakee, IL), was prepared in synthetic CSF or pooled human CSF. Calibration curve samples and QC samples were prepared independently and immediately prior to enzymatic digestion. QC sample concentration was calculated by correcting for the endogenous protein concentration derived from an appropriate pooled human CSF blank.

Enzymatic digestion of human CSF samples was automized and carried out on an Agilent Bravo liquid handling system (Agilent Technologies, Santa Clara, CA) in PP-Microplate 96 well U-shape plates (Greiner Bio-One, Kremsmünster, Austria). A starting sample volume of 10 μL was used for generating the calibration curve, QC and study samples. A master mix solution of internal standards (10 μL) was added to each sample. This solution consisted of synthetic, heavy-isotope labeled surrogate peptides (Biosynth, Lelystad, the Netherlands) to serve as internal standards for assay normalization: (H₂N–NPANPVQ–OH (MW: 744.8 Da, Hp 1-1), H₂N–DYAEVGR–OH (MW: 818.8 Da, Hp). Prior to digestion, a protein denaturation step was carried out at 70 °C for 20 min after the addition of 50 μL of a 1% sodium deoxycholate (DOC) solution (BioXtra, Sigma-Aldrich, St. Louis, MO) and 90 μL of 50 mM ammonium bicarbonate (Seppro, Sigma-Aldrich). Following sample denaturation, samples were allowed to cool down prior to enzymatic digestion. Digestion was performed by adding 30 μL of a trypsin solution (1 μg/μL) (Trypsin from porcine pancreas, Sigma-Aldrich, solubilized in 1 mM HCl from Merck KGaA, Darmstadt, Germany). Enzymatic digestion was performed by incubating samples at 37 °C for 1 h followed by quenching the reaction by the addition of 10 μL of 10% formic acid solution (Thermo Scientific, Waltham, MA) and incubation at 25 °C for 10 min. Following digestion, samples were centrifuged at 4000 rpm for 15 min using an Eppendorf Centrifuge 5810 (Eppendorf, Hamburg, Germany). 100 μL of the supernatant was then transferred into a new 96-well plate for immediate LC-MS/MS analysis. Sample volume required for analysis was 1 μL.

LC-MS/MS analysis was conducted on an Acquity UPLC M-Class system (Waters, Milford, MA) coupled to a QTRAP 6500+ mass spectrometer (Sciex, Framingham, MA). Chromatographic separation method was achieved using a 15 min gradient at 60 °C with a nanoEase™ M/Z Peptide BEH C18 (300 Å, 1.7 μm, 300 μm × 100 mm) column (Waters) at a microflow rate of 10 μL/min, using as mobile phase A water with 0.1% formic acid and mobile phase B acetonitrile with 0.1% formic acid (both LC-MS grade, VWR Chemicals, Radnor, PA). An MRM acquisition method was employed on a QTRAP 6500+ mass spectrometer (Sciex) to monitor specific transitions for both the endogenous (light) surrogate peptides and their heavy-labeled internal standards. The instrument was operated in positive ion mode. The Optiflow 1–50 µl Micro Source was used for analysis. Source parameters were set as follows: ion spray voltage of 4500 V, ion source gas 1 set at 30.0, ion source gas set to 0.0, curtain gas set at 30.0, collision gas set to medium, temperature set to 0.0, unit resolution for both Q1 and Q3. Parameters specific to each peptide’s transition were individually optimized, including declustering potential, entrance potential, collision energy and collision exit potential. Detailed information on the MS1 parent-ion targets and their MS2 product-ion transitions provided in Supplementary Table S1. The instrument’s valve was diverted to waste between minute 0.0–4.5 and 10.5–15.0, while MRM acquisition was scheduled between minute 4.5–10.5. MS raw data was acquired in Sciex Analyst software (Version 1.7.2) and processed using Sciex OS software (Version 2.0). Absolute quantification was achieved by comparing the MRM area ratios of the surrogate peptides of each protein of interest to their corresponding heavy labeled isotopologue peptides serving as internal standard. The absolute protein concentrations were calculated via the comparison on these area ratios to those in the established calibration curve. Calibration curves were constructed by using peak area ratios of endogenous peptide signals to internal standards with a weighted (1/x2) least-squares linear regression analysis.

### Animal studies

All animal experiments were performed in accordance with Swedish legislation. The experimental procedures have been evaluated and approved by the local ethical committee in Malmö/Lund, ethical approval Dnr 5.8.18-06020/2019.

The preterm rabbit pup model was used as previously described,^26,33^ with cesarean section performed on gestational day 29 (E29, term 31–32 days), a developmental stage considered approximately equivalent to 24–28 weeks of human gestation.^27,34^ Pregnant New Zealand White does (Charles River, France, and Egle Kergiene, Lundsbrunn, Sweden) were anesthetized with intravenous Propofol-Lipuro (20 mg/mL i.v., B. Braun Medical AB, Danderyd, Sweden) prior to cesarean delivery. After delivery, the pups were handled and nursed by animal laboratory staff. The pups were dried and placed in a cage placed in an infant incubator (30 °C and 60% humidity). Pups were hand-fed twice a day (ColoDan Whole Colostrum, Biodane Pharma, Gesten, Denmark and Fox Valley 30/50, Melk voor Dieren, Rotterdam, the Netherlands), starting at 100 mL/kg, and increasing daily (10 mL/kg) up to maximum 140 mL/kg, using a 4 French feeding tube (Vygon, Ecouven, France). Feeding at 12, 36, 60 and 84 h of age was reduced to half of the previous corresponding dose, i.e., 50 mL/kg at 12 h of age.

### Experimental setups

In the first part of the study, a total number of 41 pups (Supplementary Table S2, 50% female, determined as described below) and 6 does (5 pregnant and 1 non-pregnant) were included. Blood samples were collected from the pregnant doe/adult rabbit in Microvette® 500 Lithium heparin LH tubes and centrifuged at 1800 x *g*, 10 min, room temperature. Supernatant was transferred to a new tube, snap frozen on dry ice and stored at −80 °C until subsequent analysis. Subsequently, the adult rabbits were sedated, and the pups were delivered within a few minutes. The doe/adult rabbits were terminated through an i.v. injection of Allfatal vet. (>1 mL/kg, Omnidea AB, Stockholm, Sweden) and CSF was collected from the neck region, fourth ventricle basal cistern, using a 27-gauge syringe, and transferred to a 1.5 mL centrifugation tube and centrifuged at 1800 x *g*, 10 min, room temperature. Supernatant was transferred and snap frozen on dry ice and stored at −80 °C. Brains were dissected out from the skull. The cerebrum was coronally sliced (approx. 1–2 mm thick), separated into hemispheres and separate samples were collected from the frontal, middle and posterior parts of cerebrum, and snap frozen on dry ice (one piece from each respective part and hemisphere) and stored at −80 °C.

At approx. 1–2 h of age, the pups were weighed, and hand-fed as described above except for animals in groups 0 and 2 h, which were euthanized directly (see Supplementary Table S2 for more details). Remaining groups were sacrificed 8–144 h after birth. At sacrifice, pups were anaesthetized with an intramuscular (i.m.) injection of Ketaminol vet. (50 mg/mL, Intervet AB, Stockholm, Sweden) and Rompun vet. (20 mg/mL, Elanco Denmark, Ballerup, Denmark) in combination with isoflurane inhalation (1000 mg/g, Virbac Carros, France). CSF was sampled as above, using a 30-gauge syringe, and prepared as described above. Blood samples were collected through heart puncture in Microvette® 500 Lithium heparin LH, and an ear biopsy (for sex determination) was collected and snap frozen on dry ice. Afterwards, pups were transcardially perfused with phosphate buffered saline (PBS tablets, Medicago, Uppsala, Sweden), containing heparin (1000 IU/mL, LEO, LEO Parma AB, Malmö, Sweden), and the brains were dissected out from the skull as described above, and snap frozen on dry ice and stored at −80 °C.

In the second part of the study, a total number of 46 pups (Supplementary Table S3, 33% female, determined as described below) were included in and cared for as described above. An intraperitoneal (i.p.) administration of 50% (v/v) sterile glycerol solution (6.5 g/kg, dose adjusted for 50% glycerol solution, BioVision, Milpitas, CA) were given using a BD Microfine + 1 mL (29 gauge) to induce IVH. Verification of IVH were performed at 24 and 72 h of age using high-resolution ultrasound (HRU, Vevo 2100, VisualSonics Inc., ON, Canada) with a MS-550D 40 MHz transducer as described in Sveinsdóttir et al.^26^ (Fig. 1b). Animals were sacrificed 24–96 h after birth as described above.

### Hb (heme) and Hp analysis of rabbit plasma and CSF

Rabbit Hp in plasma and CSF samples was determined using the Rabbit HP/ Haptoglobin ELISA Kit (LifeSpan BioSciences, Seattle, WA). Samples were diluted first in PBS (PBS tablets, Medicago) and then in sample diluent and analyzed according to user manual. Absorbance was measured (450 nm) with a plate reader (Infinite F50, Tecan, Männedorf, Switzerland) and processed with Magellan data analysis software (Magellan™ 6.6 for Infinite 50, Tecan) and Microsoft Excel (Microsoft Corporation; Redmond, WA).

Heme was quantified in rabbit plasma and CSF with the QuantiChrom Heme Assay Pup (DIHM-250; BioAssay Systems, Hayward, CA) according to instructions from the manufacturer. Absorbance was measured (405 nm) with a plate reader (Infinite F50, Tecan) and processed with Magellan data analysis software (Magellan™ 6.6 for Infinite 50, Tecan).

### Sex determination of rabbit pups

Rabbit sex was determined by confirming the presence of the sex determining region of the Y gene (SRY, gene ID: 100328958) in the rabbit genome using PCR and visualized by gel electrophoresis as previously described.^33^

### Statistical analysis

Statistically significant differences and applied statistical tests used are presented in the respective figures and figure legends. Values of *p* < 0.05 were considered significant. All statistical calculations were performed with GraphPad Prism (GraphPad Prism 9.4; GraphPad Software; GraphPad, Bethesda, MD).

## Results

### Hp and Hb levels in CSF of preterm infants with IVH

**Fig. 2:**
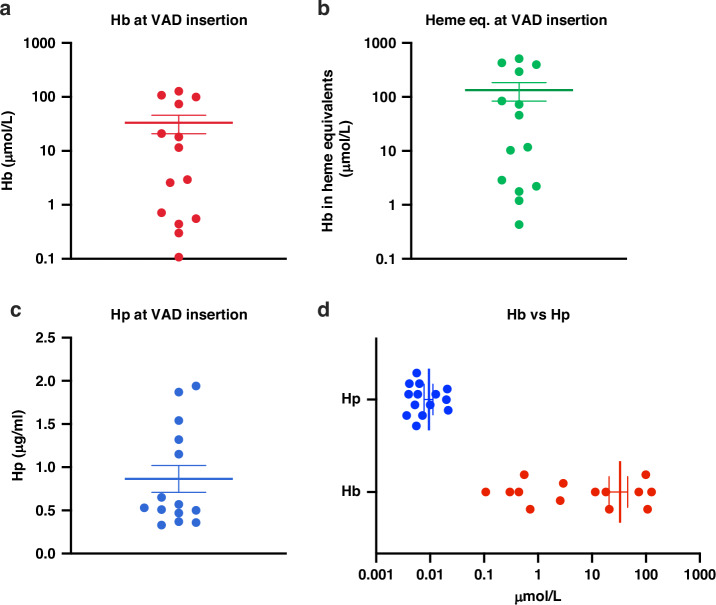
Hp and Hb concentrations in the CSF of infants with IVH at insertion of VAD. Levels of Hb (**a**), levels of Hb in heme equivalents (**b**) and levels of Hp (**c**). A comparison of molar concentrations of Hb and Hp respectively on a logarithmic scale (**d**). Data is presented as individual values with mean ( ± SEM).

In the preterm infants (Table 1), a reservoir (ventricular access device, VAD) was inserted to enable CSF withdrawal, which occurred at a mean ( ± SD) age of 19.4 ( ± 12.0) days, while samples for measurements of Hb and Hp were taken at an age of 22.7 ( ± 13.3) days. Intraventricular CSF concentrations of cell-free Hb in the infants were 33.3 ± 12.5 µmol/L (mean ± SEM; Fig. 2a), corresponding to 133.3 ± 50.1 µmol/L when expressed in heme equivalents (Fig. 2b). The mean CSF concentration of Hp was 0.87 ± 0.15 µg/mL (corresponding to approximately 10 nmol/L, Fig. 2c). The data suggests that the scavenging capacity is very low at insertion of the reservoir, with the molar ratio of Hb concentration being more than 3000 higher than that of Hp (33.3 µmol/L Hb, while 0.87 µg/mL Hp ≈ 10 nmol/L, Fig. 2d).

**Table 1:** Clinical characteristics of IVH infant cohort

Infant characteristics	Mean/number
Number of infants	14
Gestational age at birth (weeks, mean ± SD)	26.6 ± 2.4
Birthweight (g, mean ± SD)	1046 ± 384
IVH III/Periventricular hemorrhagic infarction (*N*/*n*)	8/6
Postnatal age at detection of hemorrhage (days, mean ± SD)	3.5 ± 3.9
Postnatal age at VAD insertion (days, mean ± SD)	19.4 ± 12.0
Sampling Time point (days after birth, mean ± SD)	22.7 ± 13.3
Sampling Time point (days after IVH, mean ± SD)	19.2 ± 14.1

### Rabbit Hp levels in adult and preterm pups

**Fig. 3:**
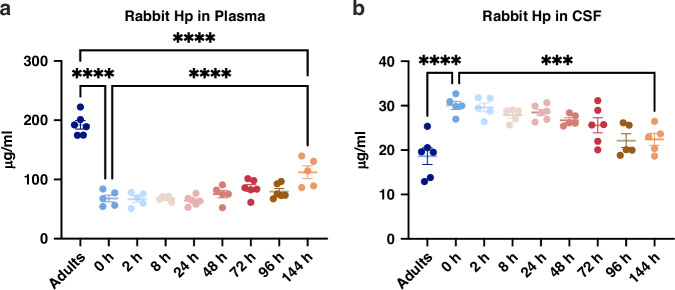
Plasma and CSF levels of rabbit Hp in preterm rabbit pups and adult rabbits. Levels of Hp in preterm rabbit pups (0 to 144 h) and adult rabbits in plasma (**a**) and CSF (**b**). Statistical comparisons were made between adults vs. pups at 0 h, between pups at 0 h vs. 144 h and adults vs. pups at 144 h. Data is presented in scatter plots with mean ( ± SEM). Statistical comparisons were calculated using one-way ANOVA with Šídák’s multiple comparisons test. ***P < 0.001, ****P < 0.0001.

To establish baseline concentrations of rabbit Hp, levels in plasma and CSF were measured in both adult and preterm pups (Fig. 3). In plasma, rabbit Hp levels were significantly higher in adults (192.3 ± 7.3 µg/mL, mean ± SEM) than in pups (0, 2, 8, 24, 48, 72, 96 and 144 h of age). The pups had an increasing trend, with the lowest levels at birth (67.9 ± 5.7 µg/mL) and highest at 144 h (112.1 ± 10.7 µg/mL). The pups, however, did not reach adult levels during this period. In the CSF, the opposite was seen with lower adult levels than in pups (Fig. 3b). Furthermore, the levels of rabbit Hp in CSF decreased in the pups from highest levels at 0 h (30.1 ± 0.9 µg/mL) to the lowest level at 144 h (22.4 ± 1.4 µg/mL), and at 144 h no significant difference was detected between pups and adults (18.6 ± 1.9 µg/mL).

### Rabbit Hp and heme levels in preterm pups with IVH

**Fig. 4:**
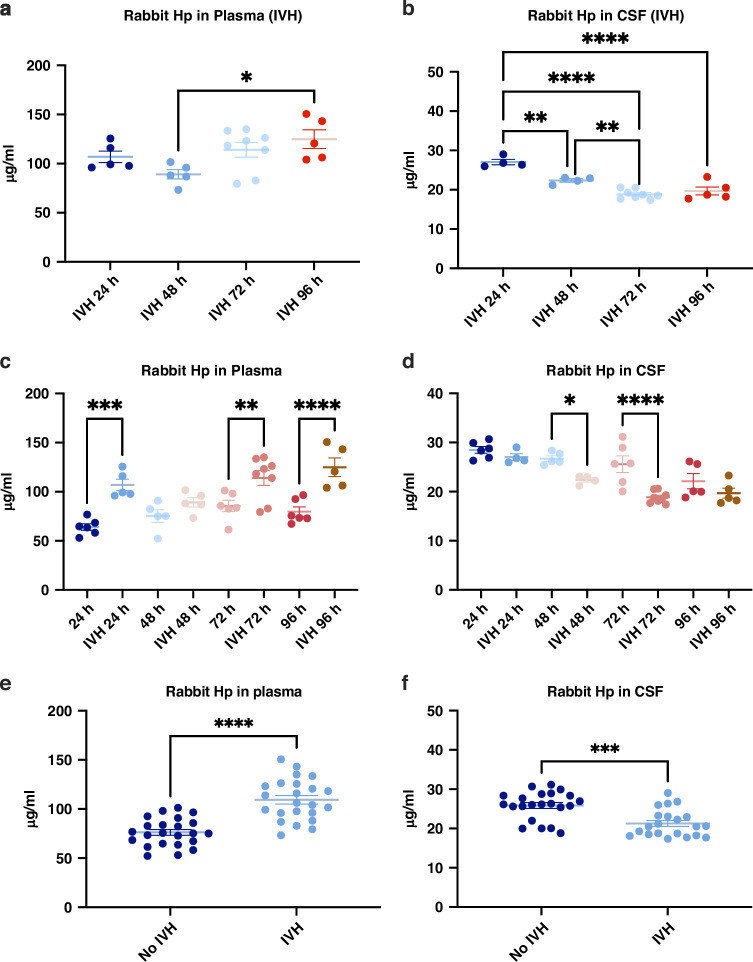
Plasma and CSF levels of rabbit Hp in preterm rabbit pups with or without IVH. Levels of rabbit Hp in plasma (**a**) and CSF (**b**) in preterm rabbit pups with IVH (24 to 96 h of age). Comparison of Hp levels between animals with and without IVH in plasma (**c**) and CSF (**d**). Pooled levels of Hp at all timepoints in plasma (**e**) and CSF (**f**). Data is presented as scatter plots with mean ( ± SEM). Statistical comparisons were calculated with a one-way ANOVA with Šídák’s multiple comparisons test (**a**–**d**) or a paired t-test (**e**, **f**). *P < 0.05, **P < 0.01, ***P < 0.001, ****P < 0.0001.

Rabbit Hp was also measured in rabbit pups with IVH from 24 h (after detection of bleeding with ultrasound) and up to 96 h of age (Fig. 4). In plasma, a similar trend as in non-IVH animals (Fig. 4a) was seen, with increasing levels from 24 to 96 h of age, although only a significant difference between 48 h (89.1 ± 4.8 µg/mL) vs. 96 h (124.9 ± 9.5 µg/mL). In CSF, a decrease over time was observed with levels decreasing significantly between 24 h (27.0 ± 0.7 µg/mL) and 48 h (22.4 ± 0.4 µg/mL), as well as between 48 and 72 h (18.9 ± 0.4 µg/mL). Thereafter, levels stabilized, showing no significant difference between 72 and 96 h (19.7 ± 1.0 µg/mL). Comparing the levels of rabbit Hp in plasma between animals with or without IVH (Fig. 4c), preterm rabbit pups with IVH had significantly higher levels at 24, 72 and 96 h. In the CSF, preterm rabbit pups with IVH had significantly lower Hp levels at 48 and 72 h of age (Fig. 4d). Combining pups with or without IVH independently of age, animals with IVH had significantly lower levels in CSF (21.29 ± 0.75 µg/mL for IVH vs. 25.85 ± 0.76 µg/mL), but higher levels in plasma (109.5 ± 4.37 µg/mL for IVH vs. 76.17 ± 2.93 µg/mL, Fig. 4e, f).

**Fig. 5:**
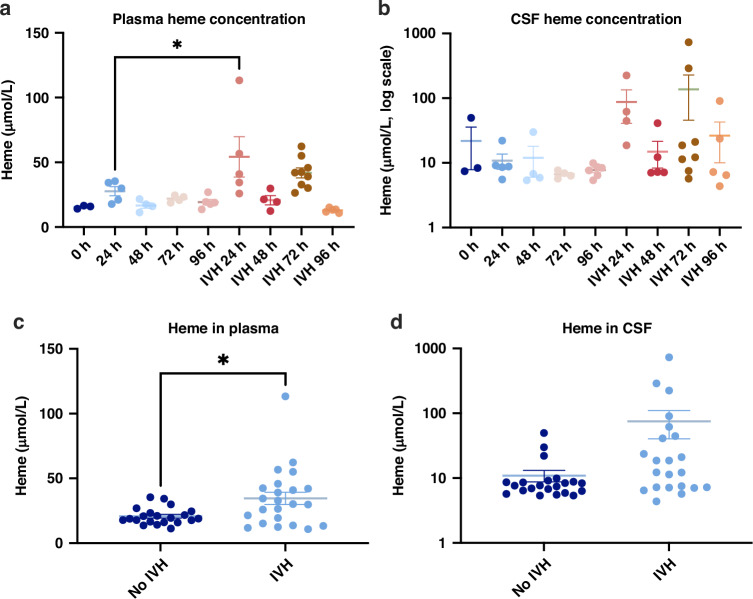
Heme levels in plasma and CSF in preterm rabbit pups with or without IVH. Levels of heme between 0 to 96 h (no IVH) and 24 h to 96 h (IVH) in plasma (**a**) and CSF (**b**). Pooled levels of heme derived from all timepoints in plasma (**c**) and CSF (**d**). Data is presented as scatter plots with mean ( ± SEM). Statistical comparison was calculated using one-way ANOVA with Šídák’s multiple comparisons test (**a**, **b**) or a paired t-test (**c**, **d**). *P < 0.05.

Direct quantification of Hb in rabbit samples was not possible due to lack of species-specific assay sensitivity. Heme was therefore measured as a proxy for cell-free Hb, a commonly used approach when direct Hb detection is limited, given the high correlation between Hb and heme concentrations in biological fluids.^35^ Although not statistically significant, a tendency towards increased levels of heme were detected in pups with IVH in CSF and with a significant increase in the plasma at 24 h (Fig. 5a, b). Furthermore, when dividing animals into IVH or no IVH (i.e. with no regard to timepoints), animals with IVH had significantly higher heme levels in plasma (34.58 ± 4.79 µmol/L for IVH vs. 20.77 ± 1.40 µmol/L for no IVH, Fig. 5c). Similarly, a clear trend towards higher heme levels in CSF were observed in animals with IVH vs. no IVH (Fig. 5d), although the difference was not statistically significant (75.21 ± 34.93 µmol/L for IVH vs. 10.95 ± 2.22 µmol/L for no IVH, *p* = 0.07). To enable an estimation of the endogenous Hb-scavenging capacity of preterm rabbit pups in the CSF (pooled animals between ages of 24–96 h), heme concentrations were recalculated to Hb equivalents by assuming that one Hb tetramer contains four heme groups (i.e. a heme concentration of 75 µmol/L corresponds to approximately 18.75 µmol/L of Hb). Given a measured CSF Hp concentration of 21.29 µg/mL, which equals approximately 0.2 µmol/L, this yields an estimated Hb:Hp molar ratio of about 100:1.

## Discussion

Cell-free Hb and its downstream metabolites have been suggested to act as mediators of inflammation and oxidation following IVH, thus constituting a significant risk of causing brain damage.^18,19^ Furthermore, it has been suggested that scavenging may reduce the detrimental effects of cell-free Hb.^36^ Based on this, we propose Hp, the main cell-free Hb-scavenger, as a potential therapeutic alternative in extremely preterm infants with IVH. In this study, we have investigated the scientific rationale of Hp as a treatment for preterm IVH by establishing an understanding of 1) the basal levels of endogenous cell-free Hb (heme) and Hp in preterm rabbit pups, and 2) the levels of cell-free Hb and Hp following IVH in extremely preterm infants and in preterm rabbit pups. Here, we observed that following IVH there are substantially higher levels of Hb in CSF compared to Hp in both extremely preterm infants and preterm rabbit pups, 3000-fold in the infants and 100-fold in the pups. This, clearly supports the hypothesis that targeting the overabundance of Hb, known to contribute to the damage seen in IVH, with a Hb-scavenging protein, e.g. Hp could be beneficial.

The plasma levels of Hp in newborns are much lower than those in adults, approx. 50 µg/mL (cord blood)^37^ vs. 300–2100 µg/mL,^38^ with subsequently increasing levels in infants from birth and onwards.^37–39^ Moreover, preterm infants have even lower plasma levels than at term.^37^ In line with humans, we observed that in rabbit plasma, the adults had significantly higher levels than the preterm pups. Furthermore, there was a rapid increase in the plasma Hp-levels over the first six days in the pups, thus displaying a comparable physiology to that observed in human infants. Similarly to humans,^40^ Hp levels in rabbit CSF were considerably lower than in plasma, but interestingly and in contrast to the humans, the preterm rabbit pups initially had higher levels of CSF Hp than their adult counterparts. Taken together, these findings indicate that rabbits show developmental patterns of Hp that parallel those in humans.

Collection of CSF samples from healthy infants is not ethically justified. However, published data from infants undergoing lumbar puncture for other clinical indications, such as screening for sepsis/meningitis, report Hp concentrations of around 15 µg/mL.^41^ In comparison, the data presented here showed substantially lower levels (mean 0.87 µg/mL). Similarly, Hb is typically absent or detectable only at minimal concentrations in non-hemorrhagic CSF. While some studies suggest that preterm infants may have a low background presence of Hb,^42^ these levels remain far below the markedly elevated concentrations observed in our IVH cohort.

In line with previous studies in preterm infants with IVH,^43–45^ we observed high levels of cell-free Hb at the insertion of the VAD in the infants with IVH. Analysis of Hb levels (heme) in the preterm rabbit pups displayed that the IVH-pups had higher levels of Hb in both plasma and CSF, although not statistically significant in the CSF. In the CSF, the variation within the IVH-group was extensive, which might be due to differences in size of bleedings and/or sampling timepoint. In this study, we did not grade the bleeding (e.g. as is performed in the infants: grade I-IV),^46^ instead we only categorized them in a binary yes or no approach, which might explain the varying levels. Moreover, due to the very small CSF volume in rabbit pups, withdrawal could only be performed once, i.e. at sacrifice. Thus, Hb levels are only measured at one timepoint for each animal and, hence, might not reflect peak concentrations. For the infants, we present data from the first CSF withdrawal after VAD insertion. Consequently, these values may not reflect peak Hb concentrations either and the wide variation observed may be influenced by the timing of sampling after IVH occurrence as well as the size of the initial hemorrhage.

In both the infants and the preterm rabbit pups with IVH there were exceedingly higher levels of Hb compared to Hp, more than 3000-fold in the infants and 100-fold in the pups. Although the rationale for enhancing the scavenging potential in the CSF is reasonable in both cases, the relative levels of Hp in infants are considerably lower. This may be due to timing, that is the VAD insertion and subsequent CSF collection was performed at a later timepoint than that of CSF sampling in the rabbit pup. As a result, more consumption of Hp may have had time to occur in the human infants. Further, compensational mechanisms related to the production of Hp in response to Hb exposure may occur at different rates in infants as compared to rabbit pups.

In the present study, brain tissue was not processed for histological analysis and no anatomical nor inflammatory evaluation was performed. However, prior studies using the preterm rabbit pup IVH model have demonstrated a colocalization of cell-free Hb with key periventricular areas, including the ventricular walls, choroid plexus, and cerebellum, but without evidence of widespread neuroinflammation at the relevant timepoints.^18,19^ Another limitation of the present study is that Hb levels could not be directly quantified in the rabbits; instead, heme was measured as a proxy. Importantly, previous studies have demonstrated a very strong correlation between Hb and heme levels when using standardized assays.^35^ In addition, analysis of infant CSF displayed almost no detectable metHb, instead oxyHb was the clear majority. This indicates that heme remained largely bound within Hb and supports the assumption that measured Hb closely reflects heme concentrations. Furthermore, as other heme proteins are present in the CSF only at very low levels, their contribution to the measured signal is likely negligible.^47–50^

To mimic the disease progression and, thereby, be able to evaluate therapeutic interventions, animal models are needed. In IVH, the glycerol-induced preterm rabbit pup model has several benefits compared to rodent models. For instance, rabbits have a more complex brain structure with a perinatal white matter maturation timeline similar to that of human infants and the model also mimics important features associated with preterm birth.^51,52^ Here we report a new aspect of similarities, i.e. high levels of cell-free Hb and low Hp, between the rabbit pup IVH-model and the preterm infant following IVH. To further emphasize the model’s translational relevance, Fig. 1 presents representative ultrasound images of a preterm infant and a preterm rabbit pup with IVH, illustrating the echogenic features used for IVH identification and the visual similarity between clinical and experimental presentations. For future studies, the growing arsenal of neurobehavioral tests for rabbits, as described by Derrick et al.^53^ will be crucial in the assessment of pharmaceutical interventions of preterm IVH as developmental complications constitute an important morbidity following IVH.

Previously, Hb measurements have been established in cohorts of preterm infants suffering from IVH,^43–45^ and our findings here align with these data, as we report comparable levels. Mahaney et al. and Strahle et al. also reported very low levels of Hp and the heme-binding protein hemopexin, and interestingly, lower hemopexin levels were associated with increased ventricle size,^41,54^ suggesting its potential as an indicator for worse outcome.^55^ This further strengthens the idea that the administration of scavenging proteins, such as Hp, hemopexin or α_1_-microglobulin, might be beneficial to diminish adverse effects on the immature brain following the hemorrhage.^56–60^ Specifically, in adults, Hp has also been suggested as a treatment for both subarachnoid hemorrhage and intracerebral hemorrhage, where exogenously added or overexpression of Hp has improved outcome in mouse models.^57,58^ This highlights an exciting next step: evaluating Hp supplementation as a treatment in a preterm IVH animal model.

In conclusion, we present data displaying that the preterm rabbit pup IVH-model mimics two fundamental aspects of disease progression in extremely preterm infants suffering from IVH; i) a high presence of cell-free Hb in the CSF, and ii) a very low cell-free Hb scavenging potential. While previous studies have demonstrated increased CSF Hb following IVH and characterized downstream injury mechanisms, our study is, to our knowledge, the first to quantify and compare the relative concentrations of Hb and its primary scavenger, Hp, in both preterm infants and a translational preterm rabbit pup model. By estimating Hb:Hp molar ratios, we highlight the limited scavenging capacity in the immature CSF environment. This cross-species comparison not only reinforces the preterm rabbit pup as a key preclinical model to evaluate Hb-scavenging strategies but also provides a clear rationale for targeting Hb clearance as a treatment approach in IVH.

## Supplementary information

Supplementary material

## Data Availability

The datasets generated during and/or analyzed during the current study are available from the corresponding author on reasonable request.
