# Supplementary material for: Exploring hemoglobin dynamics and scavenging mechanisms in preterm infants and preterm rabbits with cerebral intraventricular hemorrhage
Source: Pediatr Res. 2025 Nov 14;99(7):2783–91. doi: 10.1038/s41390-025-04556-2 (PMC13485674; doi:10.1038/s41390-025-04556-2)
Supplement: Supplementary file 1 [file 41390_2025_4556_MOESM1_ESM.pdf]

Supplementary material - Exploring Hemoglobin Dynamics and Scavenging Mechanisms in Preterm Infants and Preterm Rabbits with Intraventricular Hemorrhage

Supplementary Table S1. Detailed information on the MS1 parent-ion targets and their MS2 product-ion transitions.

Q1 mass (Da)	Q3 mass (Da)	Dwell Time (sec)	ID	DP (volts)	EP (volts)	CE (volts)	CXP (volts)
405.19	531.29	40.00	DYAEVGR_Hp_+2y5_light	97.00	12.00	17.00	11.00
405.19	460.25	40.00	DYAEVGR_Hp_+2y4_light	95.00	13.00	19.00	11.00
405.19	279.10	40.00	DYAEVGR_Hp_+2b2_light	94.00	13.00	19.00	11.00
410.20	541.30	40.00	DYAEVGR_ Hp_+2y5_heavy	94.00	13.00	20.00	11.00
410.20	470.26	40.00	DYAEVGR_ Hp_+2y4_heavy	95.00	13.00	19.00	11.00
410.20	279.10	40.00	DYAEVGR_ Hp_+2b2_heavy	97.00	12.00	17.00	11.00
370.19	593.30	40.00	NPANPVQ_ Hp1_1_+2b6_light	50.00	10.00	12.00	11.00
370.19	147.08	40.00	NPANPVQ_ Hp1_1_+2y1_light	50.00	12.00	16.00	11.00
370.19	283.14	40.00	NPANPVQ_ Hp1_1_+2b3_light	50.00	10.00	18.00	11.00
373.20	599.32	40.00	NPANPVQ_ Hp1_1_+2b6_heavy	35.00	13.00	11.70	11.00
373.20	147.08	40.00	NPANPVQ_ Hp1_1_+2y1_heavy	37.00	10.00	14.00	11.00
373.20	397.18	40.00	NPANPVQ_ Hp1_1_+2b3_heavy	39.00	13.00	20.00	11.00

Supplementary Table S2. Number of rabbit pups at each timepoint.

Group	N=	Termination (h)
1. **Preterm Pups – 0 hours**	4	0
2. **Preterm Pups – 2 hours**	5	2
3. **Preterm Pups – 8 hours**	5	8
4. **Preterm Pups – 24 hours**	6	24
5. **Preterm Pups – 48 hours**	5	48
6. **Preterm Pups – 72 hours**	6	72
7. **Preterm Pups – 96 hours**	5	96
8. **Preterm Pups – 144 hours**	5	144
9. **Adult Rabbits**	6	-

Supplementary Table S3. Number of rabbit pups with or without IVH at each timepoint.

Group	N=	Glycerol (g/kg)	Route of glycerol adm.	IVH	Termination (h)
1. **Preterm Sham Control**	3	0	-	-	0
2. **Preterm Sham Control**	5	6.5	i.p.	No	24
3. **Preterm Sham Control**	4	6.5	i.p.	No	48
4. **Preterm Sham Control**	4	6.5	i.p.	No	72
5. **Preterm Sham Control**	6	6.5	i.p.	No	96
6. **Preterm IVH**	5	6.5	i.p.	Yes	24
7. **Preterm IVH**	5	6.5	i.p.	Yes	48
8. **Preterm IVH**	9	6.5	i.p.	Yes	72
9. **Preterm IVH**	5	6.5	i.p.	Yes	96
